# Successful long-term maintenance of *Mansonella perstans* in an in vitro culture system

**DOI:** 10.1186/s13071-017-2515-8

**Published:** 2017-11-10

**Authors:** Abdel Jelil Njouendou, Manuel Ritter, Winston Patrick Chounna Ndongmo, Chi Anizette Kien, Gandjui Tchamatchoua Victor Narcisse, Fanny Fri Fombad, Dizzle Bita Tayong, Kenneth Pfarr, Laura E. Layland, Achim Hoerauf, Samuel Wanji

**Affiliations:** 1 Parasite and Vector Research Unit (PAVRU), Department of Microbiology and Parasitology, University of Buea, Buea, Cameroon; 2 Research Foundation for Tropical Diseases and the Environment (REFOTDE), Buea, Cameroon; 3 Institute of Medical Microbiology, Immunology and Parasitology, University Hospital Bonn, Bonn, Germany; 4 German Centre for Infection Research (DZIF), Bonn-Cologne partner site, Bonn, Germany

**Keywords:** *Mansonella perstans*, In vitro culture, Worms, L3 infective larvae, Long-term maintenance

## Abstract

**Background:**

Approximately 114 million people are infected with *Mansonella perstans* in large proportions of Africa. In contrast to other filariae that infect humans, *M. perstans*-infected individuals show no distinct pathology or specific clinical picture, indicating a well-tuned adaptation to the host. In addition, since *M. perstans* adult worms reside in serous cavities which are difficult to access, research has been hindered and there is a paucity of knowledge about the biology of *M. perstans*, especially the development of the different life stages as well as *M. perstans*-driven immune responses. Thus in this study, an in vitro culture system was developed which allows an in-depth analysis of *M. perstans*.

**Results:**

*Culicoides* species were caught in Ediki (Kumba), Southwest Region within Cameroon following a blood meal on a microfilaremic donor that had 1500 microfilariae/ml of peripheral blood and kept in captivity for 12 days at 23 °C. In a pilot experiment, 15 infective larvae were obtained from the midges and co-cultured with a confluent monolayer of monkey kidney epithelial cells (LLC-MK2) in DMEM medium supplemented with 10% FBS for up to 77 days. The resulting survival rates of 33% revealed that the cell-conditioned medium was suitable for long-term maintenance of *M. perstans* worms. To confirm these preliminary observations, 249 infective larvae were cultured for 50 days and their development was monitored daily and microscopically graded for motility. In total, 170 (68.3%) filariae survived and 124 (49.8%) larvae moulted between days 21–30 to become L5 stage larvae which were motile and showed continuous vigorous movement.

**Conclusion:**

We have established an in vitro culture system for the generation and long-term maintenance of viable *M. perstans* worms. This technique will be an important tool to study parasite biology and development, the role in host immunity, and might be helpful to discover novel treatment strategies against this filariae.

**Electronic supplementary material:**

The online version of this article (10.1186/s13071-017-2515-8) contains supplementary material, which is available to authorized users.

## Background

*Mansonella perstans* is a well-adapted filarial nematode and, unlike in lymphatic filariasis (LF) or onchocerciasis, infection causes no distinct pathology with only mild symptoms like subcutaneous swellings, skin rashes and pleuritis [1, 2]. It is estimated that 110 million people in over 33 countries, especially in the tropical parts of Latin America as well as large portions of Africa are infected [1, 2]. The transmission of *M. perstans* requires a bite of a midge of the genus *Culicoides* [2], but in many endemic areas, the specific *M. perstans*-transmitting *Culicoides* species remains unclear. Upon transmission, the infective larvae (L3) develop into adult worms in an unknown manner and reside in serous body cavities [1–3]. Since *M. perstans* worms have only been recovered on rare occasions, research about the development of the different life stages remains limited. However, fecund female worms can release numerous microfilariae (Mf), which circulate in the peripheral blood and can be taken up by another *Culicoides* midge during a blood meal [1–3]. Although *M. perstans* Mf circulate during day and night in the peripheral blood [4], weak diurnal periodicity with a maximum Mf intensity during the first 12 h of the day has been observed [5]. In summary, many facets of *M. perstans* infection such as the development of life stages and transmission are still unclear. Thus being able to culture *M. perstans* in vitro would provide an essential tool in unravelling many of the open questions about this filarial nematode.

Several studies have reported in vitro cultures of *Wuchereria bancrofti* [6–8], *Onchocerca* [9–12] and *Brugia* species [13–15] that have also been used to test potential anti-filarial treatment strategies [11, 16, 17]. In this study, we have established an in vitro culture system to generate *M. perstans* worms from infective larvae. Overall, 249 infective larvae, obtained from 16 different collection batches of *Culicoides*, were co-cultured with monkey kidney epithelial cells (LLC-MK2) in DMEM medium supplemented with 10% FBS. High larvae survival rates of 68.3% revealed that the applied co-culture system was suitable for long-term maintenance of *M. perstans* for up to 50 days. Moreover, 49.8% of the larvae (*n* = 124) moulted between days 21–30 of culturing to become L5 stage larvae which were vigorously motile.

## Methods

### Isolation of infective *M. perstans* larvae

*Mansonella perstans* infective larvae (L3) were obtained from *Culicoides* midges following a blood meal on a consenting donor from Ediki village (Kumba health district) that had a peripheral microfilariae (Mf) load of 1500 Mf/ml. After 15–20 min, midges were collected via aspiration into a net in darkness. Midges were then aspirated using bright torch light into tubes. This process was repeated several times between 6 pm and 6 am. Engorged midges were kept in 50 ml tubes filed one-fourth with plaster of Paris which formed a cement layer and helped to retain moisture for 12 days at 23 °C, the time required for the development of L3 from Mf in the vector. Midges were fed daily with cotton gauze soaked in autoclaved 15% sucrose solution suspended over the rim of moist paper. At intervals of 24 h, a drop of distilled water was added to the plaster of Paris to keep the gauze moist. After 12 days, L3 were isolated in dissecting medium containing RPMI-1640 medium (Sigma-Aldrich, Munich, Germany) supplemented with a 2% antibiotic cocktail (pencillin-streptomycin-neomycin; Thermo Fisher Scientific, Schwerte, Germany) using a dissecting microscope (Motic, Wetzlar, Germany). The head, the thorax and the abdomen were separately placed in three different dissecting wells containing the same dissecting medium. Infective larvae were allowed to migrate out of the various parts, especially the proboscis. The infective larvae were collected in sterile manner and parasites were washed 4 times with sterile dissecting medium and used to culture *M. perstans* worms in vitro.

### In vitro culturing of *M. perstans* worms

In 48-well flat bottomed plates (Greiner Bio-One GmbH, Frickenhausen, Germany), 1 ml of Dulbecco’s Modified Eagle Medium (DMEM; Thermo Fisher Scientific, Schwerte, Germany) supplemented with 10% foetal bovine serum (FBS; Lonza, Verviers, Belgium), 5 μg/ml ciprofloxacin hydrochloride and 10 μg/ml fluconazole or 1% penicillin-streptomycin-neomycin (PSN) (Sigma-Aldrich) was used for the in vitro culture of *M. perstans*. Ten L3 were cultured in a 48-well plate (Thermo Fisher Scientific) containing a confluent monolayer of monkey kidney epithelial cells (LLC-MK2; LGC Standard GmbH, Wesel, Germany) which were cultivated 2 days before the addition of L3. Four to ten infective larvae (average of 6–7 larvae/well) were cultured for at least 50 days at 37 °C and 5% CO_2_ and were monitored daily using a Motic AE21 inverted microscope (Motic, Wetzlar, Germany). Additionally, growth of the worms was recorded using a digital camera (Fiber Tech PC & Mobile, Putsebocht, Netherlands). Helminth viability was assessed on a daily basis via microscopy using a 4 point score sheet: 0, no movement; 1, intermittent shaking of head and tail; 2, sluggishness (movement of whole worm on one spot); and 3, vigorous movement. Mortality was defined as the percentage of dead worms in each well. An immotile worm was declared dead after observing its immobility for 5 min. Moulting and motility were scored for individual worms by two experienced trained parasitologists and results were analysed as an aggregate. The culture medium was replenished at 5 day intervals or as soon as observable colour changes were seen in the wells.

### Data processing and analysis

Data were collected daily on record sheets and entered into a template designed on Microsoft Office Excel 2007 (Microsoft, Redmond, USA). Graphs were created using PRISM 5 for Windows (GraphPad Software, Inc., La Jolla, USA).

## Results

### Establishment of a cell-conditioned in vitro culture system for the maintenance of *M. perstans* worms

**Fig. 1:**
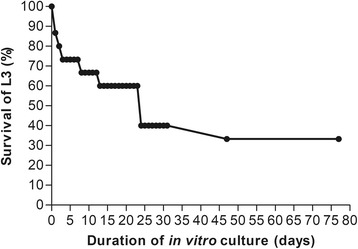
In vitro long-term maintenance of *M. perstans* infective larvae co-cultured with monkey kidney epithelia cells (LLC-MK2). *M. perstans* infective larvae (L3; *n* = 15) were co-cultured with monkey kidney epithelia cells (LLC-MK2) in DMEM medium supplemented with 10% FBS at 37 °C and 5% CO_2_ for 77 days. Cultures were monitored microscopically to determine survival rates (%) of *M. perstans* worms

Due to the inaccessibility of adult *M. perstans* worms, investigations into the developmental stages and metamorphosis of infective larvae into adult worms have not been possible until now. Therefore, we developed an in vitro culture to generate viable *M. perstans* worms from infective larvae (L3). Several studies previously described that cell-conditioned media, especially co-cultures with monkey kidney epithelial cells and inactivated FBS, were critical components for the maintenance of filariae in vitro [8–13]. Based on those studies, we established cultures of monkey-kidney epithelial cells in DMEM medium supplemented with 10% foetal bovine serum and successfully cultured *Loa loa* in vitro (unpublished findings). Based on these observations, we hypothesised that this culturing method could also be suitable for the development and growth of *M. perstans* infective larvae. Indeed, long-term maintenance of infective *M. perstans* larvae was observed under these culture condition with 40% (*n* = 6) worm survival after 31 days and 33% (*n* = 5) surviving for up to 77 days in the in vitro culture (Fig. 1).

### Development of viable *M. perstans* worms from infective larvae (L3) in vitro

**Fig. 2:**
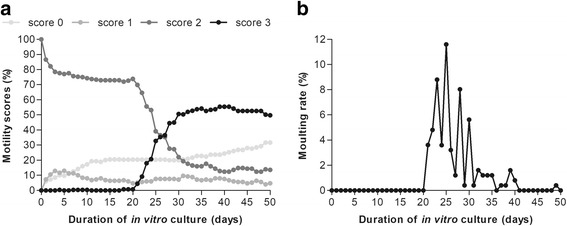
*Mansonella perstans* infective larvae moult and developed into viable and motile worms in vitro. *M. perstans* infective larvae (*n* = 249) were co-cultured with monkey kidney epithelia cells (LLC-MK2) in DMEM medium supplemented with 10% FBS at 37 °C and 5% CO_2_ for 50 days. The cultures were monitored on a daily basis via microscopy to determine (**a**) worm motility (%) according to a 4 point scale [0, no movement; 1, intermittent shaking of head and tail; 2, sluggish (shaking of the whole worm on a spot); and 3, vigorous movement] and (**b**) moulting rates (%) of the developing *M. perstans* worms

Since we were able to maintain worms in co-culture with LLC-MK2 cells, we used the co-culture to analyse the metamorphosis and motility of L3 in more detail. Thus, 249 infective larvae were extracted from 16 *Culicoides* batches following a blood meal on an *M. perstans* Mf + individual and cultured as described in the Methods section. During the 50 day culturing process, the viability and activity of each nematode was assessed on a daily basis by visual observation until they died. The motility and survival of the developing worms was monitored using a 4 point scoring system as described above. All infective larvae recovered after dissection had a starting score of 2 (Fig. 2a). After 50 days of in vitro culture, 31.7% (*n* = 79) of the worms were immotile (score 0), but overall, 68.3% (*n* = 170) survived (score 1–3). In addition, the activity of the worms with score 3 (49.8%, *n* = 124) increased over the period of the in vitro culture, whereas the percentage (13.7%) and number (*n* = 34) of worms with sluggish movement (score 2) decreased. Interestingly, 60.2% (*n* = 150) of the larvae moulted and became L5 stage larvae between days 21–30 (*n* = 127, Fig. 2b), which corresponded to the increase in viability observed through score 3. Furthermore, videos recorded on day 20 (Additional file 1), 30 (Additional file 2), 40 (Additional file 3) and 50 (Additional file 4) show the development and growth of *M. perstans* worms over the time of in vitro culture. To get an impression of the worm size, we randomly obtained five worms from the in vitro culture and measured the length. Interestingly, the worms had lengths of 40–60 mm (mean = 48.4 mm, median = 47 mm; SD = 8.2 mm) after 50 days of in vitro culture.
**Additional file 1:** *Mansonella perstans* worms after 20 days of in vitro culture. On day 20 of the in vitro culture, motility of *M. perstans* worms were microscopically monitored and recorded at 10× magnification. (MP4 1737 kb)

**Additional file 2:** *Mansonella perstans* worms after 30 days of in vitro culture. On day 30 of the in vitro culture, motility of *M. perstans* worms were microscopically monitored and recorded at 10× magnification. (MP4 2282 kb)

**Additional file 3:** *Mansonella perstans* worms after 40 days of in vitro culture. On day 40 of the in vitro culture, motility of *M. perstans* worms were microscopically monitored and recorded at 10× magnification. (MP4 4426 kb)

**Additional file 4:** *Mansonella perstans* worms after 50 days of in vitro culture. On day 50 of the in vitro culture, motility of *M. perstans* worms were microscopically monitored and recorded at 4× magnification. (MP4 6039 kb)

## Discussion

The silent nature of *M. perstans* with regards to clinical symptoms and pathology development has resulted in a shortfall on studies on the biology, transmission and host evasion tactics of this nematode. Interestingly *M. perstans* is, however, considered one of the most prevalent human diseases in tropical areas with approximately 114 million infected people [1, 2, 18]. Most of the knowledge about *M. perstans* infection has been obtained as a by-product of studies with other filarial or parasitic infections [19–22] that were considered to be more important for public health. However, *M. perstans*-induced symptoms such as subcutaneous swellings, abdominal pain, skin rashes, pericarditis and pleuritis [1, 2] have been reported. Moreover, initial studies have shown that *M. perstans* infection may influence the efficacy of Bacillus Calmette-Guérin (BCG) vaccination against tuberculosis as well as the susceptibility and disease course of HIV, tuberculosis and malaria [23–26]. Those studies increased the focus on *M. perstans* research but, until now, investigations have been hindered by the inaccessibility of *M. perstans* adult worms [1–3]. Therefore, in this study we established an in vitro culture system to decipher unresolved questions about the biology and development of *M. perstans* worms. To the best of our knowledge, we demonstrate for the first time that cultures of monkey kidney epithelial cells (LLC-MK2) in DMEM medium supplemented with 10% FBS allow long term-maintenance of *M. perstans* worms for up to 77 days. These data confirm previous studies showing that cell-conditioned and inactivated FBS are critical for the maintenance of filariae in vitro [8–13]. In addition, further experiments have shown that the in vitro survival and maintenance of *Loa loa* infective larvae was improved in DMEM medium compared to RPMI and that LLC-MK2 cells are indispensible for parasite moulting (unpublished findings). Collectively, these data suggest that *Loa loa* and *M. perstans* may share similar in vitro requirements for growth and development. In addition, most of the *M. perstans* infective larvae moulted between days 21–30 to become L5 stage larvae which continued to show vigorous movement upon 50 days of in vitro culture. It is known that moulting behaviour and life duration of the different larval stages varies strongly between different filariae and indeed, there are two groups of filaria based on the duration of L3 stages in their mammalian host [27]. The first group including filariae such as *Onchocerca volvulus* and *O. lienalis* is characterized by an early moult (less than 3 days) whereas other filariae like *L. loa*, *Wuchereria bancrofti* and *Brugia* spp. execute the first moult only after a week. In this latter group, the second moult (L4 into L5) occurs significantly before day 50 [27, 28]. *Mansonella perstans* therefore seem to belong to the first group which execute an early moult from L3 into L4 stage larvae within the first 3 days of in vitro culture. Thus, it is likely that the first moult remains unnoticed or was not obvious during this study and the moult observed after 20 days marks the development from L4 into L5 stage larvae. However, in-depth analysis needs to be performed to decipher *M. perstans* moulting behaviour and the development of the different life stages in vitro in greater detail. Interestingly, the worms had lengths of 40–60 mm after 50 days of in vitro culture and are thus comparable to *M. perstans* worms (35–80 mm) isolated from infected human individuals [29]. Nevertheless, further investigations need to be performed to discriminate gender and decipher morphology and fertility of the cultured *M. perstans* worms in more detail. In addition, the in vitro culture system will allow us to elucidate the presence and role of *Wolbachia* in this filarial nematode as previous studies have reported contradictory findings showing either the absence [30, 31] or the presence of the endosymbiont in *M. perstans* worms [32–34].

## Conclusions

Collectively, these findings present a novel tool for *M. perstans* research and an approach to elucidate several unresolved questions about the biology and development of *M. perstans* life stages. These include studies about worm fertility and morphology, genome and transcriptome analysis with single sex adult worms, as well as in-depth analysis of the role of *Wolbachia* in *M. perstans* worms using immunohistochemistry, multilocus sequence typing, and treatment with anti-wolbachial antibiotics. Finally, the in vitro worm culture also provides a platform to study potential treatments against this ivermectin non-susceptible filariae [35, 36].

## Abbreviations

DMEM: Dulbecco’s modified eagle medium
FBS: Foetal bolllvine serum
HIV: Human immunodeficiency virus
L3: Infective larvae (third stage larvae able to infect human hosts)
Mf: Microfilariae
